# Chromosomal Distribution of PcG Proteins during *Drosophila* Development

**DOI:** 10.1371/journal.pbio.0040170

**Published:** 2006-04-20

**Authors:** Nicolas Nègre, Jérôme Hennetin, Ling V Sun, Sergey Lavrov, Michel Bellis, Kevin P White, Giacomo Cavalli

**Affiliations:** **1**Institute of Human Genetics, Centre national de la recherche scientifique (CNRS), Montpellier Cedex, France; **2**Centre de Recherche en Biochimie Macromoléculaire, CNRS, Montpellier, France; **3**Department of Genetics, Yale University School of Medicine, New Haven, Connecticut, United States of America; Harvard UniversityUnited States of America

## Abstract

Polycomb group (PcG) proteins are able to maintain the memory of silent transcriptional states of homeotic genes throughout development. In *Drosophila,* they form multimeric complexes that bind to specific DNA regulatory elements named PcG response elements (PREs). To date, few PREs have been identified and the chromosomal distribution of PcG proteins during development is unknown. We used chromatin immunoprecipitation (ChIP) with genomic tiling path microarrays to analyze the binding profile of the PcG proteins Polycomb (PC) and Polyhomeotic (PH) across 10 Mb of euchromatin. We also analyzed the distribution of GAGA factor (GAF), a sequence-specific DNA binding protein that is found at most previously identified PREs. Our data show that PC and PH often bind to clustered regions within large loci that encode transcription factors which play multiple roles in developmental patterning and in the regulation of cell proliferation. GAF co-localizes with PC and PH to a limited extent, suggesting that GAF is not a necessary component of chromatin at PREs. Finally, the chromosome-association profile of PC and PH changes during development, suggesting that the function of these proteins in the regulation of some of their target genes might be more dynamic than previously anticipated.

## Abstract 2

The authors map the GAGA factor and Polycomb group proteins PC and PH to *Drosophila* chromosomes. PC and PH exhibit dynamic localizations over time at loci encoding transcription factors frequently important for development.

## Introduction

In *Drosophila,* Polycomb- and trithorax-group proteins (PcG and trxG) are chromatin components that maintain the transcriptional state of the homeotic genes after it is initially set up in the early phases of embryogenesis [ 1]. This initiation step is performed by a cascade of maternal and zygotic transcription factors such as the products of pair-rule, gap, and segmentation genes. This cascade defines the transcriptional pattern of homeotic genes along the antero–posterior axis [ 2]. The majority of these transcription factors disappear at mid-embryogenesis, but the transcriptional state of homeotic genes is maintained throughout life, due to the action of PcG and trxG proteins. PcG proteins maintain the repressed state of homeotic genes while trxG factors maintain their active state.

PcG proteins act as multimeric complexes. The ESC/E(Z) complex [ 3, 4], also known as PRC2/3 complex, deposits a methylation mark on the Lysine 27 of Histone H3 [ 4]. This methylation mark is recognized by the chromo domain of PC [ 5, 6], a stoichiometric component of the PRC1 complex [ 7], which also contains the proteins PH, PSC, and dRing/Sce. PRC1-mediated gene silencing might involve contacts between these proteins and the RNA polymerase II general transcriptional machinery [ 8, 9], ubiquitination of Histone H2A [ 10], or chromatin condensation [ 11]. These functions are mediated by specific DNA regulatory sequences named PcG response elements (PREs) [ 12].

The PcG proteins PH and PC are characterized by an identical distribution on polytene chromosomes, associating to more than a hundred cytological loci [ 13]. However, only few PREs have been molecularly analyzed. The Bithorax complex (BX-C) locus contains some of the best characterized examples *(Fab-7, Mcp, iab-2, bxd)* [ 12, 14– 17]. *Fab-7* is an example of a well-studied PRE-containing element [ 18, 19] that regulates the homeotic gene *Abdominal-B* in the appropriate segments of the fly. *Fab-7* is able to recruit PcG proteins in transgenic constructs and to induce silencing of a reporter gene, as well as to act as a trxG response element (TRE) that can maintain the memory of active chromatin states induced by transcriptional activators during embryogenesis [ 19]. Three additional PREs have been described in the Antennapedia Complex locus, regulating the Hox genes *Antennapedia, Sex combs reduced* and *proboscipedia* [ 20– 23], and two PREs have been identified in the *ph-p - ph-d* gene locus [ 24]. The *engrailed (en)* [ 25], *invected (inv)* [ 26], *hedgehog (hh)* [ 27], *cubitus interruptus* [ 28], and *Cyclin A (CycA)* [ 29] genes also contain PREs. These PREs show little sequence homology, with the notable exception of the presence of consensus sites for the GAGA factor (GAF) [ 23], the product of *pleiohomeotic* (PHO) [ 30, 31], Zeste (Z) [ 8, 32], and Dsp1 [ 33] proteins. PHO and Dsp1 are PcG recruiters [ 33– 35]. Z and GAF might play a role both as PcG and trxG recruiters [ 19, 34, 36]. The presence of clustered GAF, PHO, and Z motifs at known PREs was used to develop an algorithm in order to obtain a genome-wide bioinformatic PRE prediction [ 37], but the results of this prediction were not compared with the actual binding profiles of PcG proteins.

In this work, we describe the chromosomal distribution of three PRE-associated proteins: PC, PH, and GAF. We combined chromatin immunoprecipiation (ChIP) with hybridization to DNA microarrays (ChIP on chip approach) [ 38, 39] that contain genomic DNA tiling paths from *Drosophila* X chromosome and Chromosome 2. The data show fundamental differences between GAF and the two PRC1 members PC and PH. GAF binds a substantially larger number of sites in the genome compared to PC and PH. Moreover, GAF has a tendency to bind to narrow chromatin regions while PC and PH are spread over larger regions ranging in size from several kb to hundreds of kb. PC and PH bind to large genes, encoding for transcription factors with multiple developmental roles. Finally, we found that the developmental binding profile of PC and PH is dynamic. New binding sites appear at late stages, while some of them—present in embryos—disappear during late development. These previously unsuspected dynamics suggest that these proteins might play other regulatory functions in addition to maintenance of embryonic patterns of gene expression during development.

## Results

### Analysis of Protein Distribution Profiles using Montpellier–Yale Microarrays

We developed a genomic *Drosophila* DNA microarray by combining a tiling path containing 2.9 Mb of the *Adh* region on the left arm of Chromosome 2 (2L) [ 40] with a tiling path covering 7 Mb of the tip of the X chromosome and with several smaller genomic regions of interest and control sequences such as known PREs of the BX-C, the *ph* and the *en* genes. This will be referred to in this paper as the Montpellier–Yale (MY) arrays (see Protocol S1 and Tables S1– S3 for a description of array assembly). Using these arrays, we analyzed binding profiles of PC, PH, and GAF at four developmental stages: embryos, pupae, adult males, and adult females. For each stage, two to three fully independent ChIP on chip experiments have been performed (an example of the hybridization result is shown in Figure S1). A series of controls allowed monitoring the efficiency of the ChIP in each sample. The PREs regulating the genes *en* and *ph,* as well as the PREs of the BX-C *Fab-7* and *Mcp,* are known to recruit the three proteins (with the exception of *Mcp,* which recruits PC and PH but not GAF), and they were used as positive controls (the enrichments found in each condition are shown in Table S4). For negative controls, we did not know a priori any DNA fragment that would not be bound by any of these factors at any developmental stage. However, from the biological data available on these proteins we reasoned that they might bind a minor fraction of the genome. Therefore, we added on to the microarrays 37 randomly selected DNA fragments that are unrelated to known PC, PH, or GAF target genes and that are located at scattered positions throughout the genome. Indeed, the overwhelming majority of the fragments from this set are not bound by any of the three proteins ( Table S4).

**Figure 1:**
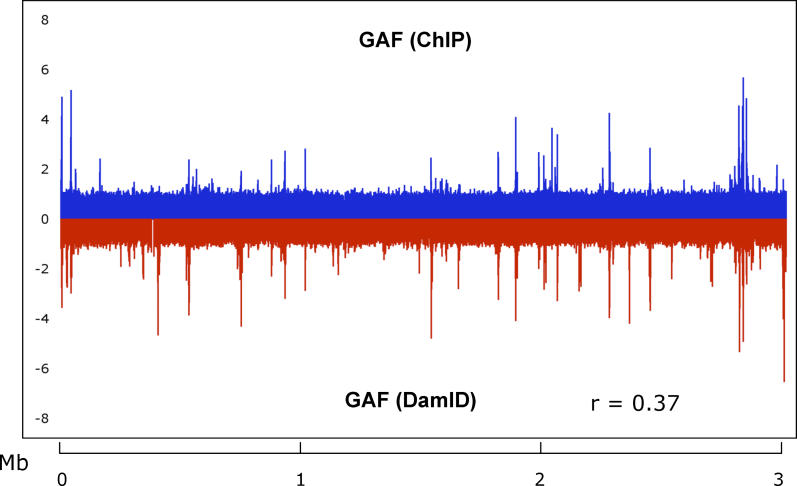
Evaluation of the ChIP Quality Comparison of protein mapping using ChIP versus DamID. The graph represents the FC of GAF binding on the *Adh* region obtained by ChIP at the embryonic stage (upper panel in blue) and with DamID in cultured Kc cells as described in [ 40] (lower panel in red). Pearson's correlation coefficient between the two distributions is indicated at the bottom of the graph.

A further validation of the ChIP profile for the GAF protein was obtained from a comparison of the ChIP on chip data with the profile already described [ 40] by using an independent method (DamID), which is based on transfection of proteins fusing the Dam methylase enzyme to the factor of interest [ 41]. The target loci are methylated by the Dam moiety of the fusion protein, and the methylated DNA fragments can be recovered and hybridized to microarrays along with a control sample where only the Dam protein is transfected. With this method, the binding profile of GAF in the same *Adh* region that was spotted on MY arrays was obtained [ 40]. The comparison between the DamID profile and the ChIP on chip profile ( Figure 1) shows a high degree of overlap with a Pearson correlation coefficient of 0.37, although the samples are obtained from different biological sources (embryos for ChIP versus cultured Kc cells for DamID).

**Figure 2:**
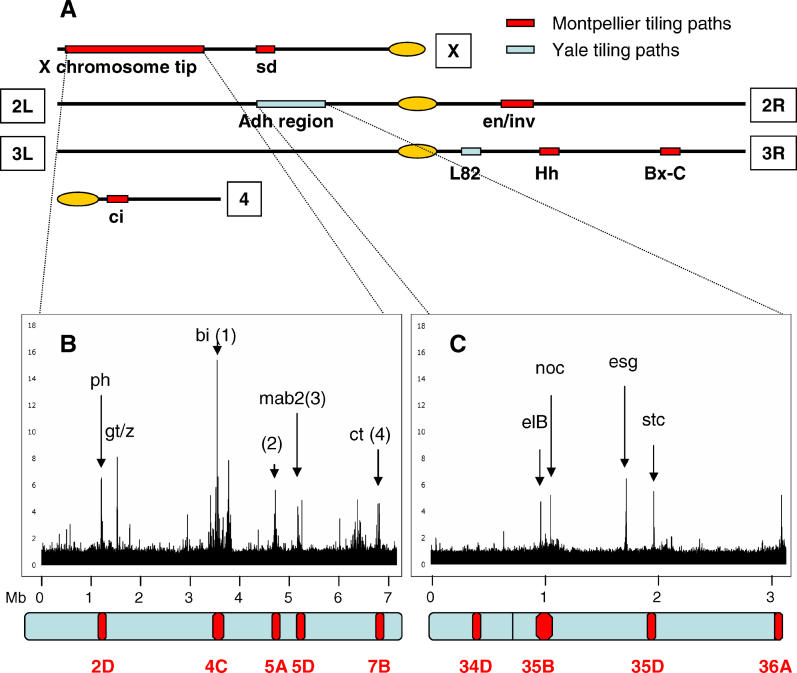
ChIP on Chip Mapping of the PH Protein Using MY Microarrays (A) Schematic representation of the four *Drosophila* chromosomes, with the Montpellier tiling path assembly shown in red, and the Yale tiling path regions shown in light blue. (B) The distribution of the PH protein along the tiling path of the X chromosome in *Drosophila* embryos. Numbers 1 to 4 indicate the regions for which FISH probes used in Figure 3 were designed. The *ph* locus is a known PcG target that served as a positive control. The other arrows point to the major binding sites. Below the graphs, a scheme of the corresponding chromosomal region is shown; with the cytological location of known PH bands in polytene chromosomes indicated as red ovals. (C) The distribution of the PH protein along the Adh region of Chromosome 2L in *Drosophila* embryos. Symbols are as in (B).

The genomic regions covered in MY arrays are schematically shown in Figure 2A. The chromosomal profile of each protein was visualized by plotting the normalized fold change (FC) between IP and control IP samples on the *y*-axis versus the position of each fragment on the *x*-axis. Figure 2 shows the chromosomal distribution of PH in embryos, as an example of the profiles that were obtained. The telomeric side of the X chromosome is shown in Figure 2B and the *Adh* region of Chromosome 2L is shown in Figure 2C (see Figure S2 for the whole set of binding profiles for each protein in each experimental condition). A large portion of the fragments was not enriched, and hybridized similarly to the control IP sample, while strong enrichments were seen at a limited number of specific domains.

**Figure 3:**
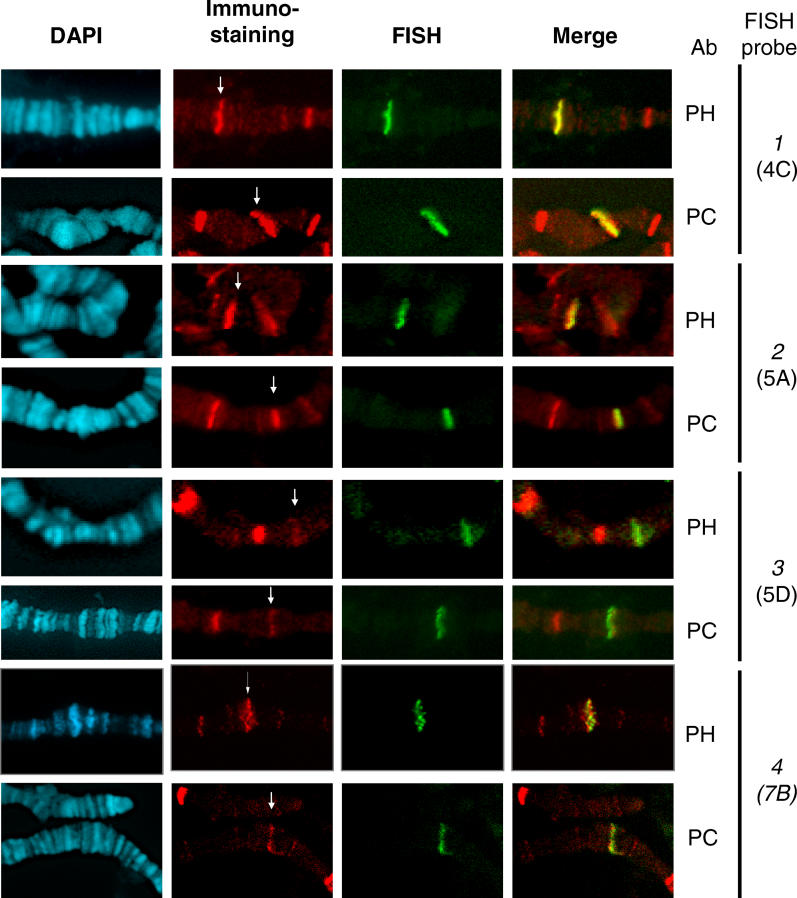
Immuno-FISH Mapping of PcG Protein Binding at Four Different Target Loci DAPI labeling of DNA is shown in light blue. The immunostainings of PC and PH (as indicated to the right of each row) are shown in red. DNA FISH staining is shown in green, and the merge of the red and the green channels is shown in right panels. The name and cytological position for each probe is indicated on the right. The numbers identifying the probes used correspond to those indicated in Figure 2. 1 corresponds to the *bifid* gene locus, 2 to the CG4136 locus, 3 to the *mab-2* locus, and 4 to the *cut* locus. The arrows point to the bands that co-localize with the FISH signal.

### Correspondence of PC/PH ChIP Sites with Cytological Mapping in Polytene Chromosomes

On the tip of the X chromosome, five PC/PH cytological binding sites were described on polytene chromosomes of third instar larval salivary glands [ 42]. These sites map at cytological locations 2D1–4; 4C1,2; 5A5,6; 5D1,2; and 7B. In the Chromosome 2L path, cytological sites 34D, 35B, 35D1, and 36A1 are bound by both PC and PH. The binding profiles from ChIP on chip at the embryonic stage show PC and PH binding at all these locations except 34D. In addition, we detected binding at locations 3A3, 4B4–6, 6D7, 7A2–7, and 35C2, which are not reported to be bound in polytene chromosomes. This indicates that the salivary gland tissue contains a subset of the total target sites, consistent with some degree of tissue specificity for PcG protein binding.

Since there is a degree of uncertainty on the location of any given polytene band in immunostaining experiments and in genomic databases, we wished to directly confirm the correspondence between the cytological location of the ChIP on chip fragments with polytene chromosome mapping. To this aim, we conducted immuno-FISH experiments on polytene chromosomes [ 43] with four different probes mapping to sites 4C1,2; 5A5,6; 5D1,2; and 7B (FISH probes corresponding to peaks 1, 2, 3, and 4, Figure 2B). These probes correspond to PC/PH binding sites located in the gene loci *bifid,* CG4136, *mab-2,* and *cut*. In all cases the FISH signal co-localized perfectly with a cytological binding site for both PC and PH ( Figure 3). Together with the correspondence between ChIP data and the polytene signal at the *ph* gene locus [ 24], this shows that the regions enriched in ChIP on chip experiments correspond precisely to the known PC/PH polytene binding sites. As a further validation of our analysis, we compared PC/PH profiles with an independent dataset, obtained from DamID experiments with the PC protein and using tiling arrays spanning the same region of Chromosome 2L that is contained in MY microarrays (B. Tolhuis, E. de Wit, I. Muijrers, H. Teunissen, W. Talhout, B. van Steensel, and M. van Lohuizen, personal communication). The distribution profiles are strikingly similar, both between PC-Dam and PC-IP as well as between PC-Dam and PH-IP. Taken together, these data show that the profiles obtained from our analysis represent the true chromosomal distribution of the proteins under study.

### Analysis of ChIP on Chip Results by the RDAM Method

To identify genomic DNA fragments that show significant enrichment for association with the proteins examined, we adapted the Rank Difference Analysis of Microarray (RDAM) method [ 44] to the ChIP on chip approach (see Materials and Methods). RDAM replaces raw signal by its rank, expressed on a 0–100 scale, which acts as a powerful normalizing procedure. Also, RDAM does not reduce replicated signals to their means, but instead only considers variations, expressed as rank differences (RDs), between individual experimental points and controls. Finally, RDAM estimates the total number of truly varying signals, assigns a *p*-value to each signal variation, characterizes the selection of a signal using the false discovery rate (FDR), in order to estimate the expected amount of false positive signals that may be present in the selected sample, and estimates the percentage of truly varying signals included in the selection (sensitivity). Sensitivity and FDR values are intimately correlated and allow to estimate the quality of each experiment [ 44]. RDAM was applied to all conditions, each represented by at least two independent experiments (see Materials and Methods, and Tables S5 and S6). We estimated the performance of RDAM using the set of positive and negative controls present in the chips. Out of 41 positive controls analyzed for the different proteins in all conditions, only one was missed by RDAM at a level of FDR ≤ 10%. By contrast, a criterion of FC ≥ 2 would have failed to detect six (i.e., about 15%) of the positive controls. Nine of the 407 negative control spots were selected as enriched by RDAM, while 14 would have been selected by an FC ≥ 2. Thus, RDAM is clearly superior to an arbitrary cutoff at FC 2. Consistent with this analysis, a substantial number of fragments enriched less than two-fold can be selected by RDAM as truly enriched in most conditions ( Table S7).

RDAM was used to filter out values that did not pass the FDR ≤10% criterion, allowing us to only plot significantly enriched signals. An example of this graphic transformation can be seen in Figure S3. Note that the major binding peaks are retained in this analysis, consistent with a high quality of the experimental data (compare Figure 2 with Figure S3, PH embryo).

### PcG Target Sites and Downstream Genes

Along the tiling paths of X chromosome and Chromosome 2L, a set of 70 DNA fragments were selected by RDAM as significantly bound by both PRC1 proteins PC and PH in embryonic chromatin. The position of these fragments in the genome allowed us to select 37 potential PcG target genes as the genes with the closest promoter ( Table 1). Sixteen of these genes code for proteins of unknown function (43%), six for enzymes (16%), one for an RNA binding protein (3%), and 14 (38%) code for transcription factors. Since only 4.9% of the total *Drosophila* genes [ 45] and 6.4% of the genes included in the X and 2L tiling paths encode for transcription factors, there is a substantial bias toward regulation of this class of genes by PcG proteins. This indicates that these factors are acting upstream of multiple transcriptional regulatory cascades.

**Table 1:**
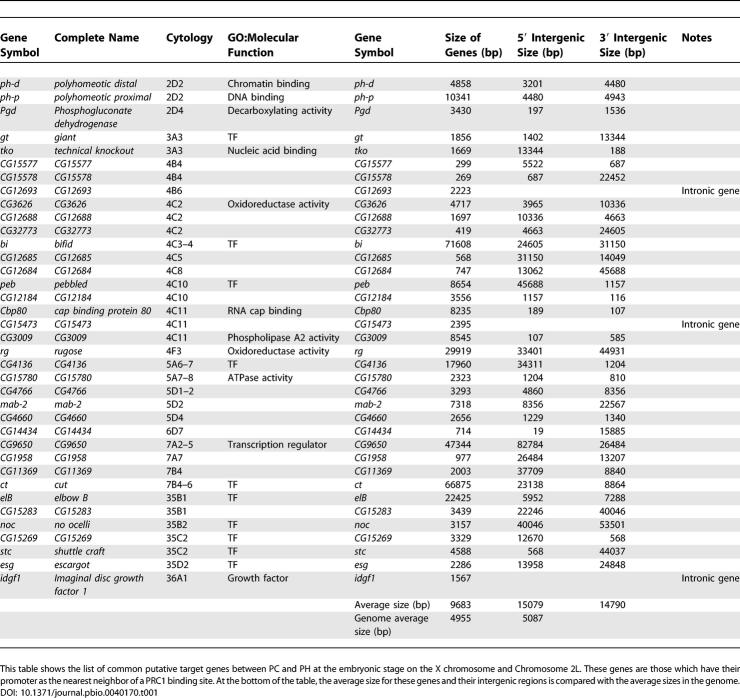
List of Target Genes for PRC1 at the Embryonic Stage

The 70 PC/PH fragments form 17 clusters of binding sites ( Table 2). We defined a cluster as a set of binding sites located adjacent to or within a series of neighboring genes, leaving no gene in the cluster without an associated PcG target DNA. The number of PcG-bound fragments within the cluster varies from one (six cases) to 23 (one case). Thus, PcG proteins are not dispersed randomly along the chromosome. Instead, they often form relatively large chromosomal domains of high PcG protein concentration. In most clusters, several bound fragments are not adjacent, suggesting that this clustered distribution does not simply reflect the presence of a single PRE from which PcG proteins spread into the flanking regions, but rather the presence of multiple PREs in each cluster. The relative distance between these clustered but spaced fragments is often in the range of 5–20 kb, similar to the interdistance of PREs along the BX-C, the only locus for which precise information is available.

**Table 2:**
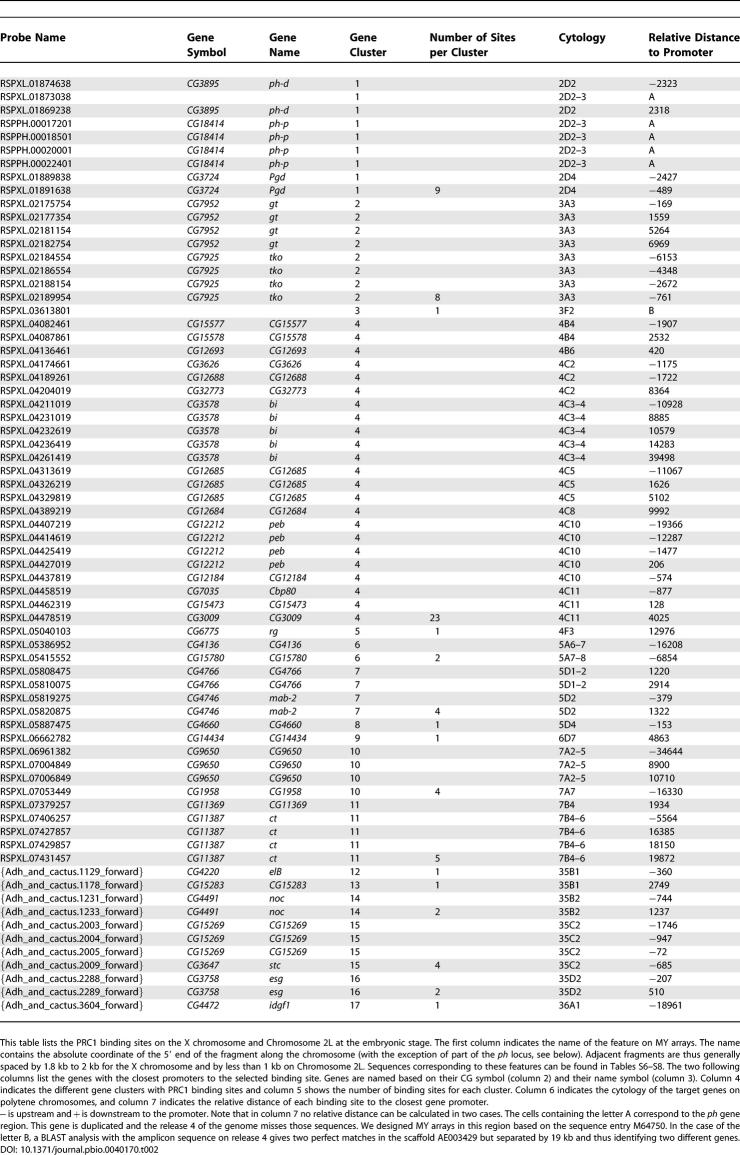
Genomic Distribution of PRC1 Binding Sites

We then analyzed the distance from the fragments significantly bound by PRC1 proteins and the promoters of the closest genes. The PREs characterized previously were sometimes found close to or overlapping the promoters of their target genes, while in other cases such as in homeotic genes they often locate to regions much more remote, up to several tens of kb away from their target promoter. This distance distribution was also found in PRC1 binding on MY arrays. 28.9% of PRC1 binding sites overlap or are located within 2 kb upstream of the 5′ end of genes ( Table 2 and Figure S4), suggesting that PcG proteins frequently associate with promoters. However, a substantial proportion (56.3 %) of PRC1 sites is located between 2 kb and 40 kb away from the closest promoter ( Figure S4). In 29.7% of these cases, the promoter-distal binding sites are not accompanied by additional PC/PH binding at the promoter of the same gene ( Table 2). The variable distance between PRC1 binding sites and promoters might reflect different modes of gene silencing, including direct obliteration of the promoter by overlapping PREs, spreading of PcG proteins from the PRE into the promoter region, or looping of PREs to reach distant promoters [ 46].

Remarkably, the sizes of the putative PcG target genes and intergenic regions are large. The candidate target genes for PRC1 on MY array have an average length of 9.7 kb, while the average in the genome is about 4.9 kb [ 47]. The intergenic regions of these genes have an average size of 15 kb, also significantly larger than the average in the genome (5 kb). These results indicate that large regulatory regions are a hallmark of PcG target genes, potentially reflecting the fact that these genes might be highly regulated at the transcriptional level.

### Developmental Dynamics of PC, PH, and GAF Chromosomal Binding

**Figure 4:**
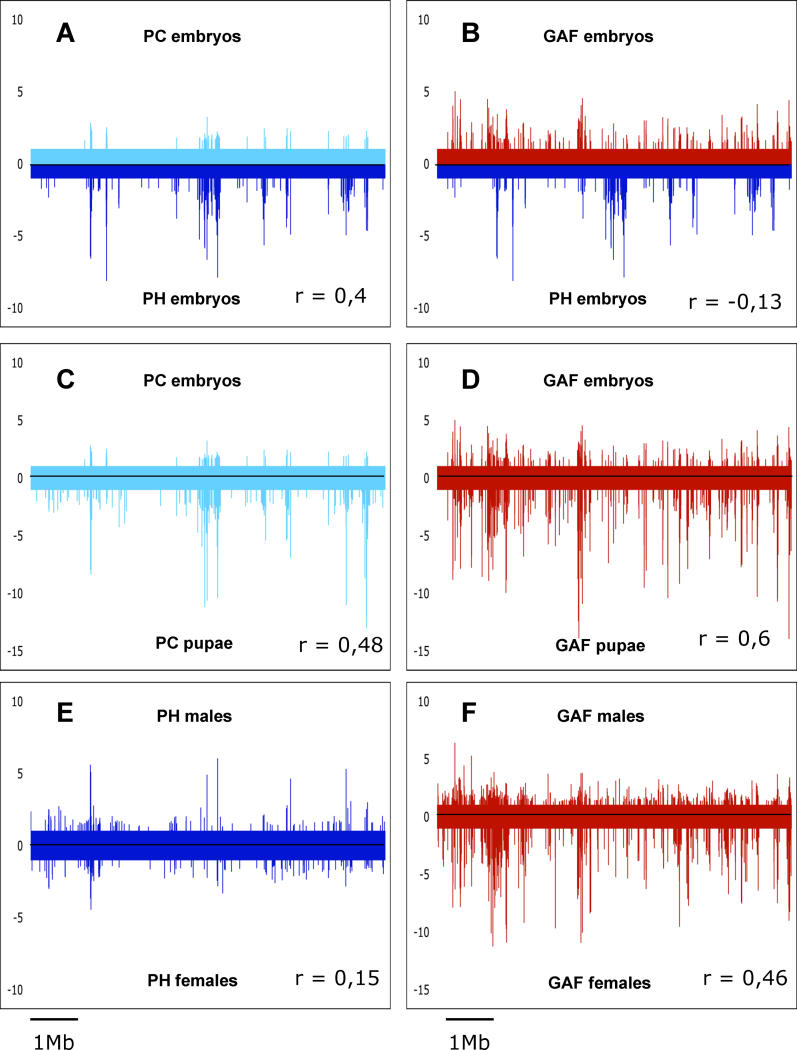
Developmental Comparison of the Distribution Profiles of PC, PH, and GAF PC is shown in light blue, PH in blue, and GAF in red. All signals that are not significantly enriched are set to one in these graphs. Thus, only the significant targets detected by RDAM at FDR 10% are shown. The correlation coefficient for each comparison is indicated above the graph. (A) A comparison between PH and PC at the embryonic stage shows the extensive overlap between the two proteins. (B) A comparison between GAF and PH in embryos shows the fundamentally different distribution profile for the two proteins. (C) Comparison between the distributions of PC in embryos over PC in pupae. (D) Comparison between the distributions of GAF in embryos over GAF in pupae. (E) Comparison between the distribution of PH males versus PH in females. (F) Comparison between the distributions of GAF in males versus GAF in females.

The binding profiles were analyzed in pair-wise comparisons to identify similarities and differences between the proteins analyzed at different developmental stages ( Figure 4 and Figure S5, Table S8). PH and PC bind the same loci on the X chromosome and even the relative binding intensities match ( Figure 4A for a comparison in embryos). Over all the MY arrays, 110 out of the 117 ChIP on chip on embryonic samples (PC E)–binding fragments are also PH E target fragments and the two profiles have a high correlation coefficient of 0.4. This observation is consistent with the perfect co-localization of PC and PH in polytene chromosomes [ 13], and with their stoichiometric co-fractionation in the PRC1 complex [ 48]. For this reason, binding of the two proteins PC and PH will be simply referred to as the PRC1 profile. In spite of previous reports that GAF is often associated with PRC1 in previously described PREs, our analysis shows that the distribution of GAF in the genome is only moderately correlated with PRC1 ( Figure 4B and Table S8). GAF binds approximately four-fold more sites than PRC1 ( Table S5) and is scattered throughout the entire tiling path, as expected based on polytene chromosome data [ 49].

**Figure 5:**
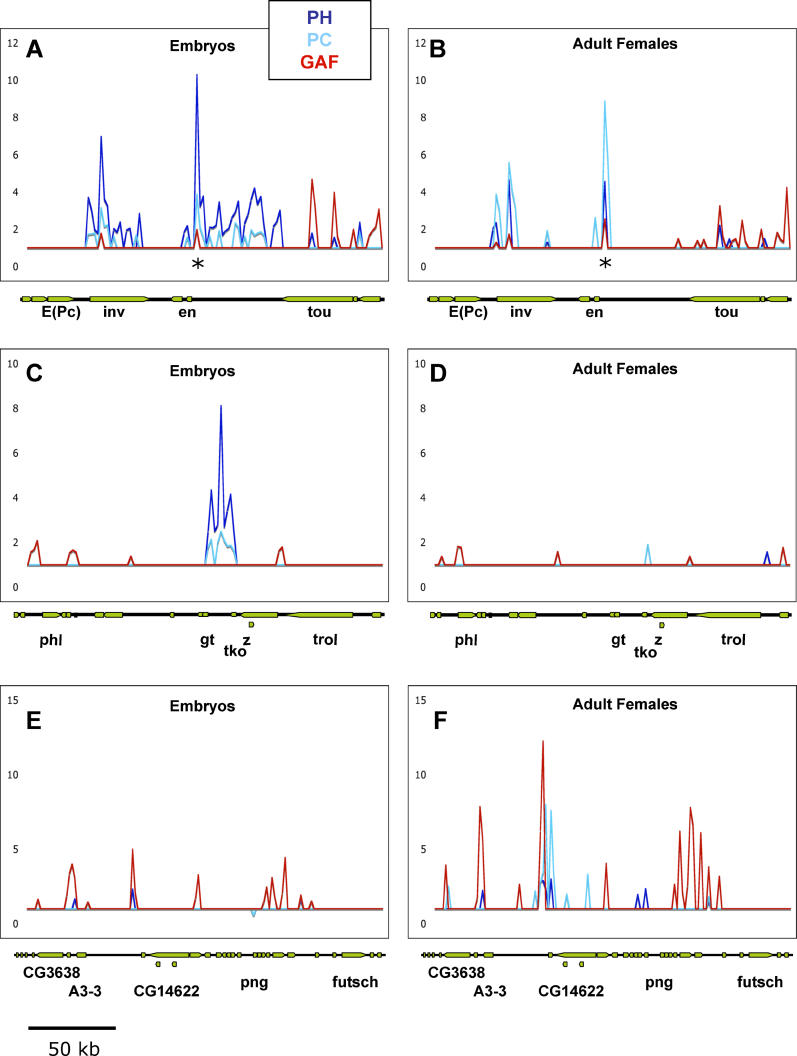
High Resolution Distribution Profiles GAF profiles are in red, PH in blue, and PC in light blue. Only significantly enriched signals detected by RDAM at a FDR of 10% are represented. Above each graph, the annotated genes of each genome region are shown. (A) Distribution profiles of PC, PH, and GAF in the *en*/ *inv* locus at the embryonic stage. (B) Distribution of PC, PH, and GAF in the *en*/ *inv* locus at the adult stage (females). The *en* PRE used as positive control is indicated by an asterisk. (C) Distribution of PC, PH, and GAF in the *gt*/ *z* locus at the embryonic stage. (D) Distribution of PC, PH, and GAF in the *gt*/ *z* locus in adult females. Note the disappearance of a strong PcG binding site, while GAF remains stable. (E) Distribution of PC, PH, and GAF in the *futsch* locus in embryos. (F) PC, PH, and GAF in the *futsch* locus in adult females. Note that a new binding site for PcG that was absent at the embryonic stage appears at the adult stage, while GAF remains stable.

The dynamics of GAF binding during development are also different from those of the PRC1 proteins. A comparison between the embryonic stage and the pupal stage for PC ( Figure 4C) shows that many PC binding sites are maintained throughout development, but major differences can be detected (see also Figure 5). These differences are seen both by comparing the embryo to the pupal stage, as well as when comparing adult males with adult females ( Figure 4E). It is interesting to note that the correlation coefficients between the distribution of PC and PH at any given stage is higher than the correlation between different stages for the same protein ( Table S8), suggesting that PRC1 association at its target chromatin is dynamic during development. Notably, the PC and PH profiles in adult males deviate most from the profiles of all other developmental stages ( Table S8), consistent with the idea that these proteins might have a specific developmental function in males [ 50].

In the case of GAF, the developmental profiles vary little from one stage to the next, as seen by the excellent correlation between the binding profiles at all stages ( Figure 4F and Table S8, and note that the correlation of GAF profiles at different developmental stages is generally higher than that of PC and PH). This suggests that this protein binds stably to most of its DNA targets throughout development, and is consistent with the high degree of overlap between GAF ChIP in animals and the GAF binding sites determined by DamID in cultured cells.

Typical patterns of distribution and of developmental dynamics of PC, PH, and GAF proteins are shown in Figure 5 at higher resolution. The three proteins associate with specific domains in the *en/inv* region, which spans 200 kb of genome and contains interesting genes such as the PcG gene *E(Pc),* the two posterior patterning genes *inv* and *en,* and the trxG gene *toutatis (tou)*. As previously described, PRC1 proteins are strongly associated with two major sites in this region [ 26]. One is just upstream of the *inv* gene and the second one spans the promoter region of the *en* gene. This second peak corresponds to the well-known *en* PRE described previously [ 25]. Moreover, the embryonic profiles of PC and PH exhibit a lower level of binding in the region flanking the two peaks, which is significantly higher than the baseline. This might reflect either the presence of previously uncharacterized PREs or a “spreading” of the two proteins away from their main PREs. Alternatively, PRC1 proteins bound stably at the major PREs might occasionally establish weak contacts with neighboring DNA fragments that might be “frozen” by the ChIP technology [ 51].

A high resolution analysis of the distribution profile of GAF shows a clear difference from PRC1. At the *en*/ *inv* locus, GAF binds the two main PRC1 peaks. This binding is functional at least in the case of *en,* as shown by mutation analysis of GAF binding sites [ 25], but it is weak compared with the strong binding seen in the flanking domain that corresponds to the *tou* gene and that is devoid of PRC1 proteins. In principle, this difference in signal intensity might depend on the different accessibility of the target chromatin to the antibodies used for ChIP. However, we believe this is unlikely since both the PC and the PH antibody seem to access the *en* region of low GAF enrichment quite efficiently. Therefore, we believe that the observed difference represents a true difference in the strength of binding. Thus, these data suggest that GAF plays roles independent from tethering of PRC1 at many loci.

While GAF stays stably associated with its targets in many cases (in Figure 5, compare left panels with right panels), PC and PH show a more dynamic profile at a number of loci. At the *gt*/ *z* locus ( Figure 5C– 5D), there is no GAF binding. In embryos, a strong PRC1 binding can be detected in a region between the *gt* and the *technical knockout (tko)* genes ( Figure 5C). Although this locus does not correspond to a polytene binding site, a regulation of *gt* by PcG proteins has been previously reported [ 52]. Binding of PC and PH is strictly limited to the region between these two genes and spans approximately 20 kb. Strikingly, however, the binding of both proteins is completely lost at all later developmental stages (see Figure 5D for an example in adult females). The reverse situation was also found. In embryos, the chromosomal domain containing the gene *CG14622* is devoid of PC and PH ( Figure 5E), while the entire region is bound by GAF on single peaks surrounding several different genes. In adult females ( Figure 5F) the binding of GAF remains stable, while significant binding is now detected for both PC and PH downstream of the *CG14622* gene.

### Regulation of *gt* and *peb* by PRC1

**Figure 6:**
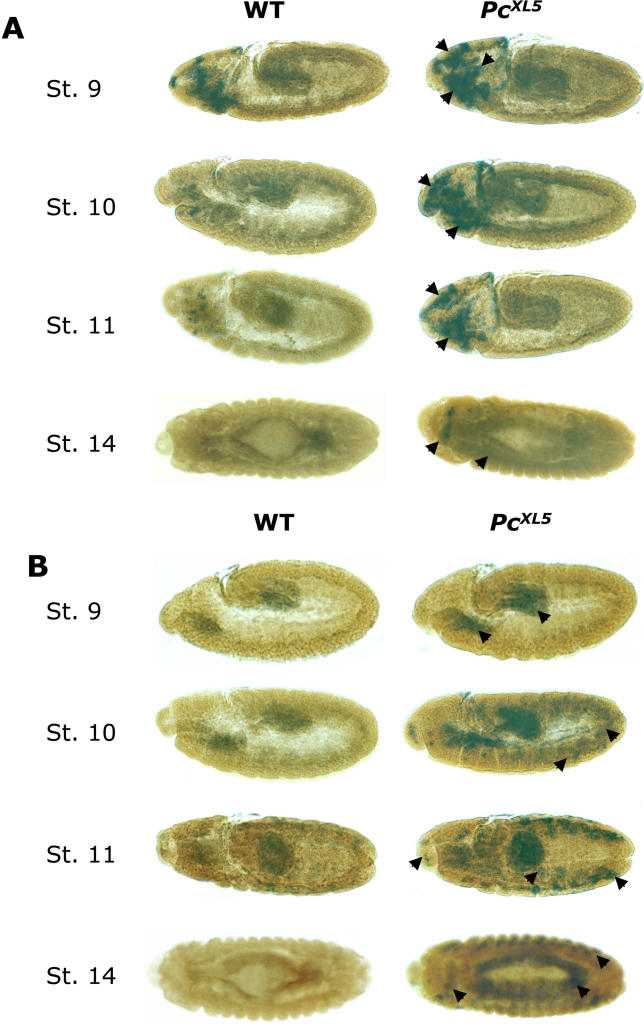
Regulation of Target Genes by PC In situ hybridizations in WT and in *Pc ^XL5^* mutant embryos for the *gt* and *peb* genes. The developmental stage of the embryos is indicated on the left. Arrowheads indicate regions of increased or ectopic labeling in *Pc* mutants compared to WT. (A) *gt* expression at embryonic stages 9, 10, 11, and 14. (B) *peb* expression at embryonic stages 9, 10, 11, and 14.

Since the *gt* locus does not correspond to a polytene binding site of PRC1, but a regulation of *gt* by some PcG proteins has been previously reported [ 52], we wished to determine whether the embryonic association of PRC1 proteins to this region corresponds to a regulatory function. We therefore analyzed the expression of *gt* in *Pc* mutant embryos by RNA in situ hybridization. To detect homozygous *Pc* mutant embryos, we analyzed a line carrying a GFP reporter under the control of the zygotically expressed *Krüppel* promoter, which allowed us to immunostain for GFP after in situ RNA hybridization (see Materials and Methods). This analysis does not allow the identification of homozygous mutant embryos before stage nine of embryogenesis. At embryonic stage nine, *gt* is expressed in part of the gnatal buds and the procephalic portion of the head. In *Pc* mutants, ectopic expression is seen in the head of all embryos (in Figure 6A, see the arrowheads). During stages 10 and 11 of embryogenesis, wild-type (WT) *gt* staining declines but persists in restricted regions of the head, until stage 14 when all staining is completely lost. In contrast, in *Pc* mutants, the head staining persists until very late stages of embryogenesis. A low level staining is consistently seen in the trunk and the abdomen in late embryos, but it is much weaker compared with the ectopic de-repression seen in the head. Thus, the ectopic expression of *gt* in *Pc* mutants is not ubiquitous, suggesting that PcG proteins are not constitutively required as repressors of *gt* expression in all tissues and developmental stages.

This result was confirmed by the analysis of *pebbled (peb),* a large gene ( Table 1) coding for a transcription factor involved in embryonic development. PC/PH binds to its promoter region as well as to two upstream regions more than 10 kb away from the promoter ( Table 2). In WT embryos, *peb* is expressed in the anterior and posterior midgut ( Figure 6B, left panels), as well as in the peripheral nervous system at stage 11. In *Pc* mutant embryos at stage 9, *peb* is overexpressed in its normal domains. Starting from stage 10, ectopic expression is additionally found in the central and the peripheral nervous systems, in the trunk and the abdominal portion of the embryo, as well as in restricted portions of the head. This ectopic expression persists at later stages, where it is particularly strong at the ectodermal rim during dorsal closure ( Figure 6B, right panels). Again, ectopic expression is not ubiquitous at any developmental stage, suggesting that the requirement for PcG-mediated silencing is restricted to a subset of embryonic cells.

## Discussion

### Establishing a Robust Dataset of Chromosomal Binding Sites for GAF and PRC1

The standardization of the experimental as well as of the bioinformatic approaches is an important component in ChIP on chip studies. Testing the ChIP samples on known gene targets by Southern blot analysis before doing the experiments on microarrays, additional validation of the GAF data by comparison with the DamID profile, and validation of PC/PH profiles by immuno-FISH on polytene chromosomes provides a strong degree of confidence in the present results. Furthermore, to improve the statistical quality of the results, we adapted the RDAM method to ChIP on chip analysis. The use of RDAM allowed estimating the sensitivity of target site detection by ChIP on chip, as well as the rate of expected false positive, defined as the FDR. This statistical analysis showed that, for most samples, a low FDR (10%) is compatible with a high sensitivity (mean of 83% and SD 15%), strongly arguing in favor of a high quality of the dataset.

### Chromosomal Behavior of PC, PH, and GAF

Together with the earlier evidence, the very good overlap between PC and PH binding at all developmental stages strongly suggests that PC and PH are stoichiometric components of one and the same biochemical complex at most of their in vivo targets and in most cells throughout development. We noticed, however, minor differences between PC and PH, particularly in adult stages. Specific analyses will be required to determine whether a subset of the targets of these two proteins is not shared.

The fact that PRC1 members bind to extended regions along the chromosome indicates that they might spread out from PREs into flanking sequences to a certain extent [ 23]. One example of this distribution is observed in the *gt* locus. The binding profile approximates a bimodal curve covering about 20 kb, and might reflect spreading of PRC1 from the peak binding site. In the case of *en*/ *inv,* PRC1 covers a region of about 60 kb. However, the binding profile differs strongly from a bimodal shape, suggesting that sequence or functional determinants may affect association of PRC1 within some chromosomal domains. Moreover, in the case of *en*/ *inv,* PRC1 binding declines sharply after a certain point, and the two genes *E(Pc)* and *tou* seem to be protected from PC/PH protein association. Since another PcG target locus, the BX-C complex, contains chromatin insulator sequences flanking PREs that might delimit their domain of action [ 53], the abrupt interruption in PRC1 binding observed in the *en*/ *inv* region might be explained by the presence of insulators at the *E(Pc)* and *tou* genes ( Figure S6).

The pattern of GAF is fundamentally different from PRC1. GAF binds to many more sites than PC and PH and it associates to DNA elements usually close to promoters of genes, consistent with the previously reported DNA binding to regions containing clustered GAGAG sites [ 40, 41]. Indeed, GAGAG sites are highly enriched among the GAF-bound fragments. They occur with an average frequency of 3.32/kb in GAF target fragments from embryos and pupae, compared with 1.34/kb in MY array sequences. Furthermore, of the 228 GAF binding fragments detected on MY arrays at the embryonic stage, only seven do not contain GAGAG motifs, confirming a strong dependence on known GAF consensus motifs for targeting in vivo. It is remarkable that, even at shared sites between GAF and PRC1, the binding profile of GAF is often distinct from PRC1. For instance, in the *en*/ *inv* region, the strongest GAF sites are in regions devoid of PRC1, although weak binding occurs at the two PREs for *en* and *inv*. Therefore, the general function of GAF is distinct from that of PRC1.

Although the role of PRC1 proteins in the maintenance of homeotic gene regulation throughout development suggests that they are stable components of their target chromatin, we observed unexpected developmental changes of PC and PH chromosomal binding. In some cases, embryonic signals were lost at later stages, while in others, binding was absent in embryos and gained during later development or in adult life. These differences suggest that the role of PcG proteins is less static than previously thought, at least in a subset of their target loci. This interpretation is consistent with the recent analysis of PcG-mediated regulation of two different target genes, *hh* and *CycA* [ 29, 54]. Both genes are direct PcG targets, but in certain tissues and developmental stages they are not bound by PcG proteins and are regulated in a PcG-independent manner. Our analysis of *gt* and *peb* expression corroborates this analysis, suggesting that PcG-mediated repression of these genes occurs only in a subset of the embryonic cells.

The strongest difference in PRC1 developmental distribution profiles was found between the embryo and the adult male stage, both for PC and PH (while the embryo and adult male profiles are highly correlated for GAF, see Table S8). Recently, a sex-specific function has been described for the HP1 protein [ 55]. Concerning PcG proteins, a specific regulation in the differentiation of the male germline has also been reported [ 50]. Our data suggest that male–female differences also exist concerning PcG-mediated gene regulation in somatic cells.

### Genome-Wide Identification of PRC1 Target Sites

We identified 17 different clusters of PRC1 binding sites potentially regulating 37 genes. Extrapolating to the whole genome, PRC1 might associate to about 200 regions and regulate approximately 400 genes at any developmental stage. By applying an algorithm named PREdictor, based on the fact that all known PREs contain clustered consensus motifs for PHO, Z, and GAF, Ringrose et al. predicted a set of 167 PREs in the whole *Drosophila* euchromatic genome [ 37]. In the two tiling paths present on MY arrays, we detected a total of 70 fragments bound by PRC1 at the embryonic stage, while Ringrose et al. predicted 14 PREs in this region. Among these 14 predictions, four fragments are found enriched in our experiments in at least one developmental stage for the PC or the PH proteins. On the other hand, 69 out of the 70 embryonic PRC1 sites from ChIP on chip do not score as PREs when run through the PREdictor algorithm. The differences between the ChIP data and the bioinformatic PRE prediction indicate that, while the presence of clustered consensus sequences for GAF, PHO, and Z is probably required for the function of many PREs, other PRE-specific sequence signatures might be needed to generate a prediction algorithm capable of systematic PRE identification. Two of these signatures might be represented by motifs that mediate PRE association of the Dsp1 and of the Grainy head proteins [ 33, 56].

### PRC1 Target Genes

The positions of PRC1 binding sites define a set of 37 genes as candidate targets of this complex. Among these genes, several functional gene categories are represented. The most striking observation is that 38% of these genes are transcription factors (TFs), a strongly overrepresented fraction when compared with 6.4% in MY arrays tiling paths. These factors regulate a variety of developmental processes, with a high proportion of them involved in embryonic patterning and in neurogenesis.

Among these putative TF targets, one interesting class is represented by repressors of segmentation and homeotic genes, such as in the case of *gt* (see also above) and of *ct*. Our data suggest that PRC1 binding is involved in the maintenance of *gt* silencing in specific regions of the head, both in stages when the gene is expressed as well as in later stages when the gene is globally shut down in WT embryos. This might suggest that PcG proteins might be involved in *gt*-dependent regulation of head patterning [ 57]. However, earlier genetic evidence indicated that several PcG genes might regulate the expression of the *gt, eve, kni,* and *cad* segmentation genes in early stages during development [ 52, 58, 59]. Although our experiments did not allow for us to test this hypothesis, a role for PcG proteins in the regulation of segmentation would be consistent with the presence of early embryonic phenotypes in *ph* mutants in addition to the classical phenotype of ectopic expression of homeotic genes [ 60].

Another regulator of homeotic genes is *ct,* which plays pleiotropic regulatory roles affecting multiple structures and cell types, among which are the nervous system, the adult muscles, and the wing margin. *ct* regulates the two homeotic genes *proboscipedia* and *Antennapedia* [ 61] and shares regulatory mechanisms with the *Ubx* homeotic gene [ 62]. The co-regulation of homeotic genes as well as of their upstream genes by PcG proteins might be an important component of developmental homeostasis. A further example of this regulatory logic is represented by the *bifid* TF gene. *bifid* is involved in regulation of wing patterning and is regulated by the *hh*/ *dpp* pathway. *hh* is itself a PcG target gene [ 27] and acts via regulation of *en* and *ci,* both of which are also PcG targets [ 25, 28]. Once again, PcG proteins co-regulate multiple steps of the same gene regulatory network.

Another class of TFs targeted by PRC1 consists of genes with a role in cell proliferation or in the coupling between cell proliferation and differentiation. In the present PRC1 dataset, they include the *escargot (esg),* the *elbowB (elB),* and the *no ocelli (noc)* genes. *esg* is a regulator of the G2/M cell cycle Cdk2/CycA and Cdk2/CycB complexes. Esg protein prevents DNA endoreplication cycles and is involved in neural and eye development. *esg* was identified in a screen for genes involved in growth and proliferation of eye precursor cells, together with *hh* and *elB* [ 63]. More detailed analysis of *elB* suggested a role in the G2 phase of the cell cycle in this tissue. *elB* and *noc* are highly conserved and interact genetically, suggesting that they might share a role in the regulation of cell proliferation in a subset of cells and developmental stages [ 64]. The function of PcG proteins in the regulation of the cell cycle in vertebrates is well-documented [ 65]. In *Drosophila,* recent studies suggest that PcG proteins regulate directly the expression of the *CycA* gene in a dynamic manner during development [ 29]. An interesting possibility is that PcG components might be involved in the regulation of cell proliferation by co-regulating multiple genes affecting this process.

In conclusion, our data suggest that PcG proteins might act as pleiotropic transcriptional regulators coordinating a variety of developmental processes. They are also consistent with recent evidence suggesting that the function of these proteins is dynamically modulated in time and space [ 29, 66, 67]. As such, it will be important to extend this analysis to the whole genome and to improve it by selecting individual cell types and following them at fine-tuned developmental stages. Since no PRE has been found yet in mammals, the ChIP on chip approach should be extended in these organisms to analyze whether PcG target sites share conserved DNA features and whether the identity of PcG target genes and of their regulatory networks are conserved in distant species.

## Materials and Methods

### MY microarrays manufacturing

Oligo design, oligo synthesis, and PCR products for the Montpellier tiling path have been provided by Eurogentec (Seraing, Belgium). The sequence used for oligo design was release 2. The sequence of the oligos and their position on the genomic assembly (corresponding to the sequence release 2), as well as the size and the sequence of each PCR amplicon, are indicated in Table S1. The oligo pairs were designed in a way that the PCR fragments have a size ranging from 1.7 kb to 2.1 kb, and adjacent fragments have different sizes. This allowed us to verify on agarose gels the size specificity and the yield of each product (See Figure S1 for an example). Moreover, randomly chosen fragments have been sequenced and blasted onto the assembled genome, and they invariably correspond to the expected amplicon. The manufacturing of the Yale tiling path is described in Sun et al. [ 40]. The sequence of the oligos, their position on the genome, and the size and sequence of PCR amplicons are indicated in Tables S2 and S3. PCR products were spotted on polylysine-coated slides by using an Omnigrid arrayer.

### ChIP experiments

ChIPs were performed on the sequenced strain of *Drosophila melanogaster,* which is *y;cn bw sp* [ 45]. Flies were grown at 25 °C on a standard medium for amplification and then the egg laying was made on plates filled with standard vinegar medium. Embryos were collected in EWB (Embryo Wash Buffer: 0.03% Triton X100, 0.4% NaCl) 12 h after the beginning of egg laying. Half of the staged embryos were used for ChIP assay and half were put back into regular medium bottles and grown until pupal stage. Pupae were then collected one day after the first pupa emerged. Again, half of the pupae were used for ChIP while the other half was grown until adult stage. Adults were then collected two days after hatching and males and females were separated for ChIP assays. The ChIP method was an adaptation of a previously published protocol [ 28]. For each ChIP, a reference sample named Mock corresponds to a ChIP performed at the same time without the addition of the specific antibody. Formaldehyde, at a final concentration of 1.8% in buffer A1, i.e., 60 mM KCl, 15 mM NaCl, 4 mM MgCl2, 15 mM HEPES (pH 7.6), 0.5% Triton X-100, 0.5 mM DTT, 10 mM sodium butyrate, protease inhibitor cocktail (Roche, Basel, Switzerland), was used for cross-linking while crushing whole *Drosophila* animals (embryos, pupae, adults) for 15 mn at room temperature. After blocking the reaction with glycine and after three washes (5 min each, at 4 °C with buffer A1), the crushed material was then filtered through Centricon Y-100 columns to recover chromatin. Subsequent steps were performed as previously described [ 28]. Sheared chromatin had an average length of 500 bp. The antibodies for IP were diluted at 1:2,000 (GAF), and 1:650 (PC and PH).

### Labeling and hybridization

1μg of both control and IP samples for each experimental condition were labeled with Cy3 and Cy5 using the Bioprime labeling kit as described [ 68]. Labeled samples were then hybridized in hybridization chambers between the microarray glass slide and a cover slip at 64 °C. After hybridization, slides were washed in subsequent steps with washing solutions with decreasing SSC concentration.

### Scanning and analysis of microarrays

Microarrays were scanned using a GenePix 4000B scanner in the two channels (Cy3 and Cy5) at the same time. The GenePix Pro 6.0 software was used to grid the microarrays and to calculate the signals for each spot. Nonhomogenous or badly conformed spots were discarded from the measurements. The raw data corresponding to scanning of each slide are shown in Tables S9– S20. The FC for each spot was then calculated after normalization to the overall median of the slide. Statistical analysis of microarray samples was performed using the RDAM method. Three completely independent ChIP samples were hybridized on MY arrays for each protein and developmental stage. In three cases (PC E, GAF E, and GAF M), hybridization of one of the samples failed. However, the two remaining samples were highly correlated when analysed with the RDAM method and, as such, these conditions could be included in the dataset. In contrast, ChIP experiments for PH in pupae failed due to unknown reasons, presumably related to the sensitivity of the antibody to chromatin prepared from pupae. Thus, this condition could not be analyzed. To eliminate further bias in RDAM analysis we performed dye swap hybridization for one of the samples in each condition. The correspondence between the dye and the samples are indicated in Tables S10 to S20.

### RDAM method for ChIP on chip analysis

We applied a modified version of the RDAM method [ 44]. In the general case, this method allows us to identify statistically significant variations of signals between two conditions C1 and C2 (a condition in our case corresponds to a ChIP from a given developmental stage using a given antibody). In a first step, each signal value was replaced by its rank in the ordered series of all the signal values, and the rank was then scaled on a 0–100 range. The scaled rank defines the relative level of each signal by its place in the overall signal distribution, and this simple transformation is a normalization method which makes all results directly comparable.

Then, each pair of signal values for a given target was converted into an RD, a measurement of variation of the signal between the two conditions, obtained by computing the difference of the corresponding ranks. To compensate for the fact that the variation is dependent upon the rank values, the following local standardization procedure was employed: zRD = RD−μ(RD)/std(RD), where μ(RD) is the local mean value of the variation and std(RD) is the local standard deviation of the variation, both calculated by moving a window (comprising 100 datapoints) across the rank range.

Finally, to assign a *p*-value to each standardized variation, we used the variation distribution observed in the case of the null hypothesis. Generally, this distribution can be constructed empirically by considering two duplicated experimental points of the same condition, e.g., C1a and C1b (no significant variation is expected when comparing biologically identical samples). This is feasible when using commercial oligonucleotide arrays that have highly reproducible amounts of product in each spot. However, duplicated experiments could not be used with our arrays because of the lower degree of reproducibility inherent to this chip technology, which uses PCR products spotted on glass slides. Instead of using two replicates, we thus constructed two series of replicates by a proper target selection in each channel. To do so, we took advantage of the fact that the binding of a protein on a target should increase, never decrease, the signal of the corresponding spot (resulting in a positive RD) when the antibody IP is compared to the control IP. When the opposite case is found, namely stronger signal in the control IP than in the antibody IP (corresponding to spots with negative RD), this does not depend on biological determinants and thus it reflects the experimental noise. The subset of spots falling in this last category could thus be used to calibrate noise. This procedure allowed us to analyze the result of one IP by comparing the antibody IP channel to the control IP channel.

Since two or three independent ChIP on chip samples were available for the same condition, the RDAM method was applied as follows. We analyzed each replicate individually, calculated a *p*-value for each of them, and combined these *p*-values into a new random variable, the product of *p*-values, which distribution in the case of the null hypothesis is easily obtained. Subsets of genes were selected by estimating the FDR, i.e., the percentage of false positives in the selection, and/or the sensitivity, i.e., the percentage of the total variation found in the selection. The zVar and the FDR values for each array feature in all conditions are given in Table S6.

The biological significance of FDR and of the sensitivity values was previously verified in the case of synthetic gene expression data [ 44], and there it was shown that the FDR assignments are slightly overestimated (symmetrically, sensitivity is slightly underestimated) by this method. Concerning the present dataset, we estimated the true sensitivity and FDR based on the controls that were included in the MY arrays. We analyzed 11 positive controls that are known to be bound by the three proteins of interest (except for the *Mcp* fragment that is only bound by PC and PH, but not GAF). This results in 41 different features that should invariably be enriched. Table S4 shows that, out of them, only one fails to be detected as significantly enriched at an FDR ≤ 10%.

It is important to note that these positive controls are not exceptionally strong binding sites (indeed, six of them fail to be detected by the FC ≥ 2 criterion). The average FC for these controls is similar as, for instance, the average value of all selected fragments for PH in embryos. Thus, the positive control sample is representative of the population of target fragments. Taken together, this suggests that the sensitivity might be slightly underestimated by RDAM. Concerning the FDR, a tentative estimation could be reached, based on the negative controls, i.e., the 37 randomly selected genomic fragments that were spotted in MY arrays. The whole analysis included 11 different conditions (different antibody and/or developmental stages), resulting in 407 individual negative control points for our test. As shown in Table S4, only nine of these points were selected by RDAM. Thus, in the worst hypothesis, 9/407 = 2.2% of the negative features were selected. This indicates that the FDR is slightly overestimated in the present analysis. Together, these data suggest that the FDR and the sensitivity are slightly underestimated by the conservative analysis that is made by the RDAM method.

### Immuno-FISH on polytene chromosomes

Immuno-FISH was performed as described previously [ 43]. FISH probes were designed using PCR fragments produced with the same primers as in MY arrays. For each PC/PH binding site to be analyzed, five adjacent PCR products were labeled with biotin (Nick translation with the BioNick kit). The PC antiserum and the affinity-purified PH antibodies were diluted 1:200 and 1:500, respectively. Detection was made by using Cy3 anti-rabbit (for proteins) and FITC anti-biotin (for FISH) secondary antibodies. Images were acquired with a Leica DMRA2 microscope and a 63× objective, and mounted with Adobe Photoshop software.

### RNA in situ hybridization

cDNAs for *gt* and *peb* were provided by DGRC. RNA probes were synthesized by incorporation of Dig-rUTP, using an RNA Transcription Kit (Stratagene, La Jolla, California, United States). The *Pc* mutant strain used for this analysis is *Pc ^XL5^,* heterozygous over the KrGFP-TM3 balancer chromosome (from stock BL#5195 of the Bloomington Drosophila Stock Center, Bloomington, Indiana, United States). Homozygous mutant embryos were identified after the in situ hybridization step by using an anti-GFP antibody (monoclonal, MMS-118P; Berkeley Antibody, Covance Research Products, Denver, Pennsylvania, United States), diluted at 1:1,000, followed by a DAB enzymatic staining (using the Vectastain kit, PK-7200; Vector Laboratories, Burlingame, California, United States). Mutant embryos have the same brownish background as WT embryos, while strains carrying one copy (heterozygous *Pc* mutants) or two copies of the KrGFP-TM3 balancer stain very strongly and appear dark brown or black.

## Supporting Information

Figure S1 PCR Quality on Agarose Gels and Example of Microarray Picture (A) An example of PCR products from the tiling path of the X chromosome checked on an agarose gel for specificity and yield. Note that adjacent products have sizes of 1.7 kb, 1.9 kb, and 2.1 kb, giving rise to a characteristic “ladder-like” pattern of migration in the gel. This allows us to easily spot fragment-size problems upon visual inspection of the gels.
(B) An example of hybridization with GAF at the embryonic stage. The background is yellowish while several dots appear red, corresponding to enriched fragments in GAF IP.
(2 MB PPT)

Figure S2 Distribution Profiles on the X Chromosome An overview of the GAGA factor, PC, and PH profiles obtained at four developmental stages (embryos, pupae, adult males, and adult females) on the X chromosome tiling path. The profiles represent an average of the normalized FC in two to three independent experiments for each protein.
(158 KB PPT)

Figure S3 Embryonic Distribution Profiles on the X Chromosome and the 2L Chromosome These graphs refer to Figure 2 but show only the significant binding sites as detected by RDAM at FDR 10%. The X chromosome is on the left and Chromosome 2L is on the right. Indicated above is the cytology of each chromosome.
(89 KB PDF)

Figure S4 Distances between PcG Target Fragments and Gene Promoters Blue shows the distribution of distances (in kb) between PcG binding sites and the closest gene promoter. − means upstream and + means downstream relative to the promoter. 28.9% of the detected PcG binding sites are located less than 2 kb upstream from the 5′ end of their closest gene, while the others are located at a distance. The distribution of distances between all fragments in the chip and the closest promoter is shown in light blue. In particular, only 14.1% of the fragments are less than 2 kb upstream of the 5′ end of the closest gene. This is significantly less than in the enriched fragment population (χ ^2^=3.9*10 ^−4^).
(152 KB PDF)

Figure S5 Unfiltered Protein Distribution Profiles Corresponding to Figure 4, but showing all FCs obtained for each experimental condition prior to filtering by RDAM at FDR ≤ 10%.
(145 KB PDF)

Figure S6 Unfiltered Protein Distribution Profiles Corresponding to Figure 5, but showing all FCs obtained for each experimental condition prior to filtering by RDAM at FDR ≤ 10%.
(338 KB PDF)

Protocol S1 Supplementary Material on the Assembly of Montpellier–Yale Microarrays (46 KB DOC)

Table S1 Montpellier Tiling Path All the features present in Montpellier tiling path, including name of the features, the sequence and physical properties of the oligos used for PCR amplification, the sequence of the amplicon, and a comment on the PCR products obtained.
(8.7 MB XLS)

Table S2 Yale Tiling Path for the Adh Region The oligonucleotide sequences and the amplicon sequences are presented for the Adh region tiling path.
(3.9 MB XLS)

Table S3 Yale Tiling Path for the L82 Region The oligonucleotide sequences and the amplicon sequences are presented for the L82 region tiling path.
(138 KB XLS)

Table S4 Positive and Negative Controls on MY Arrays Positive (upper part) and negative control sequences present on MY arrays. In each case we show two tables: top, the FDR (expressed as percentage values divided by 100) are given for each point. Bottom, the zVar values are shown, as calculated by the RDAM method [ 44] for each experimental point. zVar gives a standardized expression of the variation between the signal rank for a given fragment upon ChIP and the signal rank of the same fragment in control IP without antibody. This value replaces the FC in RDAM analysis. Its main advantage is that the normalization takes into account the absolute signal levels, eliminating problems for weak signals, where a high FC is sometimes not significant due to high standard deviations that are always associated with detection of weak signals. Highlighted in orange are the fragments detected as significantly enriched at an FDR ≤ 10%. All the positive controls were detected except in one case. Consistent with *Mcp* being a target of PC/PH but not GAF, this fragment was negative for GAF binding. The large majority of the negative controls are not detected with the RDAM method and those that are detected are generally seen in only one condition, suggesting that these signals might represent false positives. Two of the fragments might be bound by GAF since they are detected in two or three different developmental stages.
(56 KB XLS)

Table S5 RDAM Analysis of the Different Experimental Points The number of enriched signals detected by the RDAM method for each experimental condition at different FDR settings. The number of selected targets and the corresponding FDR at an estimated sensitivity of 99% [ 44] are also indicated. Please note that, for each condition, the sensitivity can be calculated as the ratio between the number of selected targets at an FDR level of 10% and the number of targets at a sensitivity level of 99%. The different datasets are indicated by the name of the protein followed by the first letter of the developmental condition studied, i.e., PC E.
(35 KB XLS)

Table S6 RDAM Analysis of MY Array zVar and FDR (expressed as percentage value divided by 100) of each probe in each experimental condition. Significant targets in our analysis are the probes with the FDR lower than or equal to 10%.
(2.4 MB XLS)

Table S7 Comparison between RDAM and FC Detection The percentage of selected fragments by RDAM for each experimental condition. The first column shows the percentage of selected fragments that have an FC < 2. The second column shows the percentage of nonselected fragments that have an FC > 2. A mean number of 36% of our selected targets have an FC < 2, and would have been rejected by the FC method, and a mean of 6% of the targets that could be rejected by the RDAM analysis have an FC > 2, and would have been selected by the FC method.
(34 KB XLS)

Table S8 Correlation of Protein Distributions in the Different Conditions Provides pair-wise comparisons between protein distributions in each experimental condition. The upper part shows the number of enriched features (selected by RDAM at a FDR of 10%) in common for each pair-wise comparison (intersecting points). In brackets is given the ratio between the observed number of intersecting points and the expected number if the distributions of the two corresponding samples were random. χ ^2^ tests were performed and they were significant with p ≤ 10 ^−7^ for all pair-wise comparisons, showing that the overlap is significant even between GAF and PRC1 proteins. The lower part shows the correlation coefficient for each pair-wise comparison. GAF distributions are generally not correlated or poorly correlated with PRC1 samples, although the χ ^2^ test shows that the points in common are higher than expected from random distributions.
(37 KB XLS)

Table S9 Targets in Common between the Different Experimental Points The intersection of common binding sites between the different conditions.
The top table shows the intersection from the RDAM analysis. In each experimental condition the fragments correspond to FDR ≤ 10%. In the top part of the table, the number of intersecting fragments for each pair-wise comparison is shown. In the bottom part, the ratio between the observed number of intersecting fragments and the number expected from the intersection of random distributions of equivalent number of fragments is shown.
The bottom table shows the result of the same analysis when, instead of using the RDAM method, all fragments with FC ≥ 2 are selected. In this case, the pair-wise intersections are still highly significant, but the ratios between observed intersection and expected intersection in the case of random distributions are much lower, suggesting that the FC ≥ 2 criterion involves a significant fraction of false positives and false negatives that can be avoided with RDAM analysis.
(37 KB XLS)

Table S10 Raw Data from Hybridization of MY Arrays Included in each file for Tables S10– S20 is the name of the experimental replicate (e.g., GAF-E-A for the first replicate of an hybridization with a GAF IP at the embryonic stage). These tables presented in Excel files are the .gpr files resulting from scanning with the Genepix scanner and present the measurements of intensity for all features on MY arrays in both channels.
(8.3 MB XLS)

Table S11 Raw Data from Hybridization of MY Arrays (13.1 MB XLS)

Table S12 Raw Data from Hybridization of MY Arrays (8.3 MB XLS)

Table S13 Raw Data from Hybridization of MY Arrays (18 MB XLS)

Table S14 Raw Data from Hybridization of MY Arrays (8.3 MB XLS)

Table S15 Raw Data from Hybridization of MY Arrays (14 MB XLS)

Table S16 Raw Data from Hybridization of MY Arrays (14 MB XLS)

Table S17 Raw Data from Hybridization of MY Arrays (14 MB XLS)

Table S18 Raw Data from Hybridization of MY Arrays (12.3 MB XLS)

Table S19 Raw Data from Hybridization of MY Arrays (13 MB XLS)

Table S20 Raw Data from Hybridization of MY Arrays (13 MB XLS)

### Accession Numbers

The National Center for Biotechnology Information (NCBI) ( http://www.ncbi.nlm.nih.gov) accession numbers are for *gt* (BT010004) and for *peb* (AY058335).

## Abbreviations

BX-C: Bithorax complex
ChIP: chromatin immunoprecipitation
*CycA*: *Cyclin A*
E: embryo
*elB*: *elbowB*
*en*: *engrailed*
*esg*: *escargot*
FC: fold change
FDR: false discovery rate
GAF: GAGA factor
*hh*: *hedgehog*
IP: immunoprecipitation
MY: Montpellier–Yale
*noc*: *no ocelli*
PC: Polycomb
PC E: PC ChIP on chip on embryonic samples
PcG: polycomb group
*peb*: pebbled
PH: Polyhomeotic
PHO: the product of *pleiohomeotic*
PREs: PcG response elements
RD: rank difference
RDAM: Rank Difference Analysis of Microarray
TF: transcription factors
tko: *technical knockout*
tou: trxG gene *toutatis*
TRE: trxG response element
trxG: trithorax group
WT: wild-type
Z: zeste
