# Supplementary material for: Chromosomal Distribution of PcG Proteins during Drosophila Development
Source: PLoS Biol. 2006 Apr 20;4(6):e170. doi: 10.1371/journal.pbio.0040170 (PMC1440717; doi:10.1371/journal.pbio.0040170)
Supplement: Supplementary file 1 [file pbio.0040170.sg003.pdf]

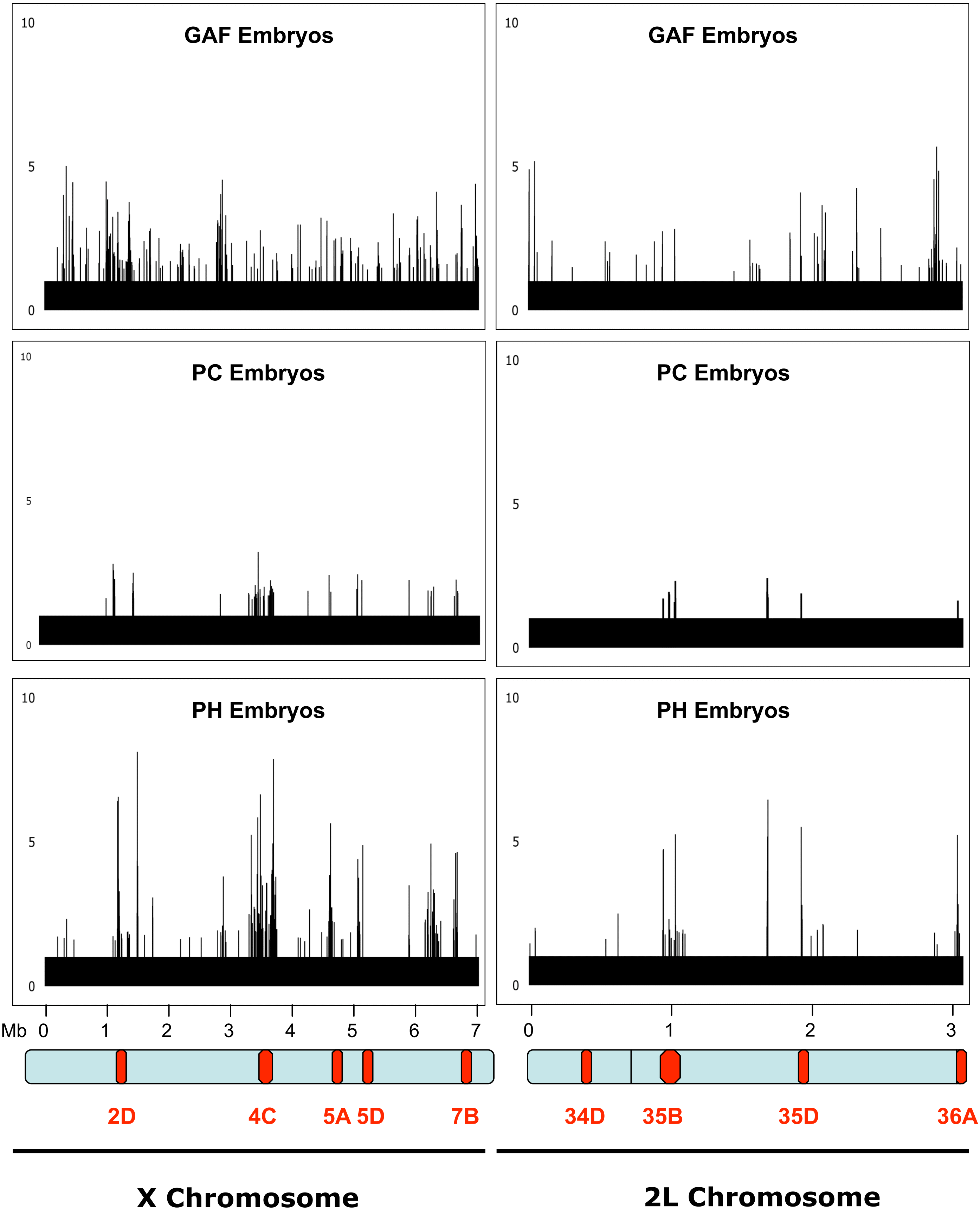

GAF Embryos
GAF Embryos
PC Embryos
PC Embryos
PH Embryos
PH Embryos
10
5
0
Mb 0 1 2 3 4 5 6 7
0 1 2 3
2D
4C
5A 5D
7B
34D
35B
35D
36A
X Chromosome
2L Chromosome

Figure S3
