# Supplementary material for: Chromosomal Distribution of PcG Proteins during Drosophila Development
Source: PLoS Biol. 2006 Apr 20;4(6):e170. doi: 10.1371/journal.pbio.0040170 (PMC1440717; doi:10.1371/journal.pbio.0040170)
Supplement: Supplementary file 2 [file pbio.0040170.sg004.pdf]

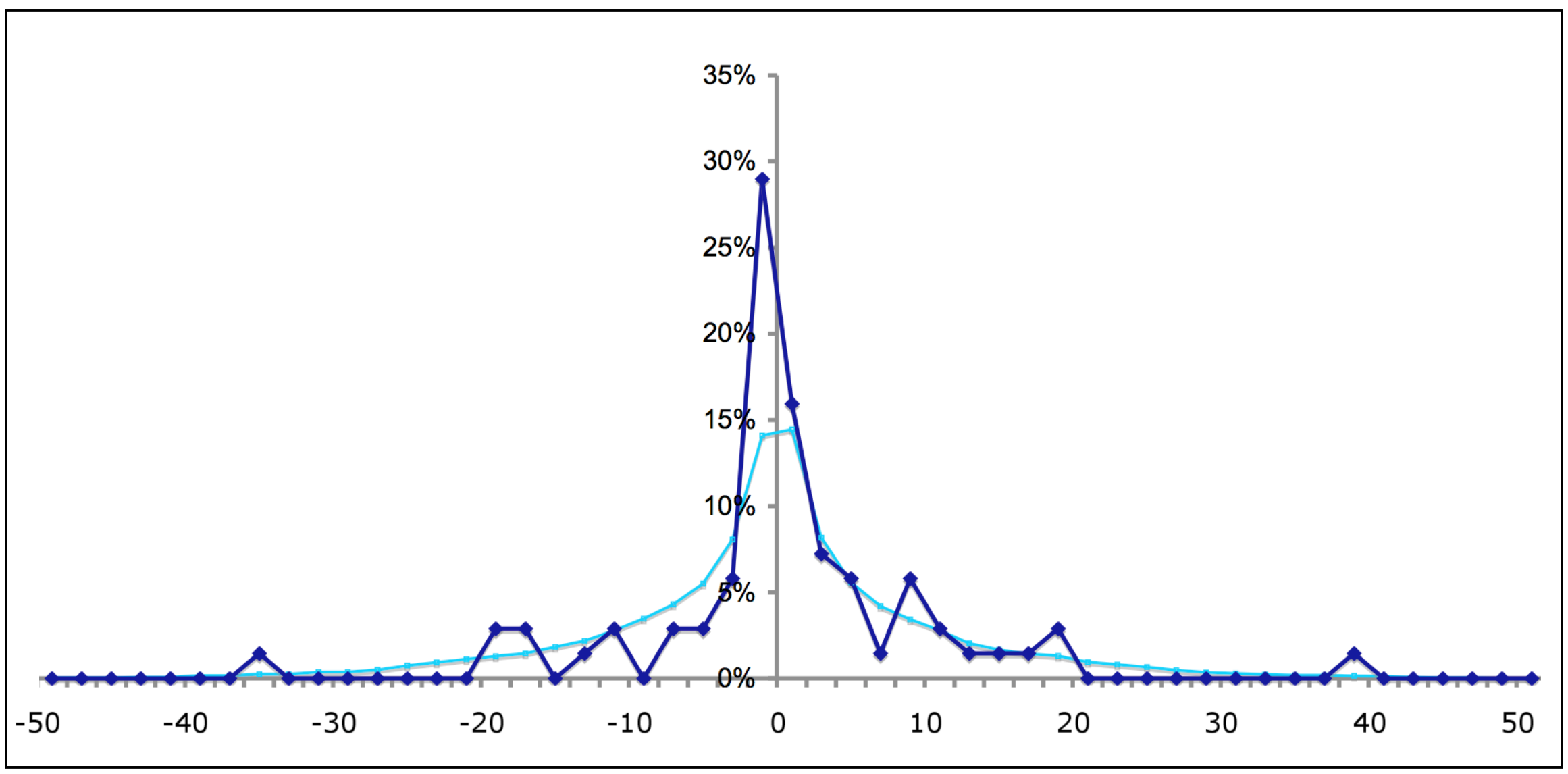

Distance in kb

Figure S4
