# Supplementary material for: Chromosomal Distribution of PcG Proteins during Drosophila Development
Source: PLoS Biol. 2006 Apr 20;4(6):e170. doi: 10.1371/journal.pbio.0040170 (PMC1440717; doi:10.1371/journal.pbio.0040170)
Supplement: Supplementary file 3 [file pbio.0040170.sg005.pdf]

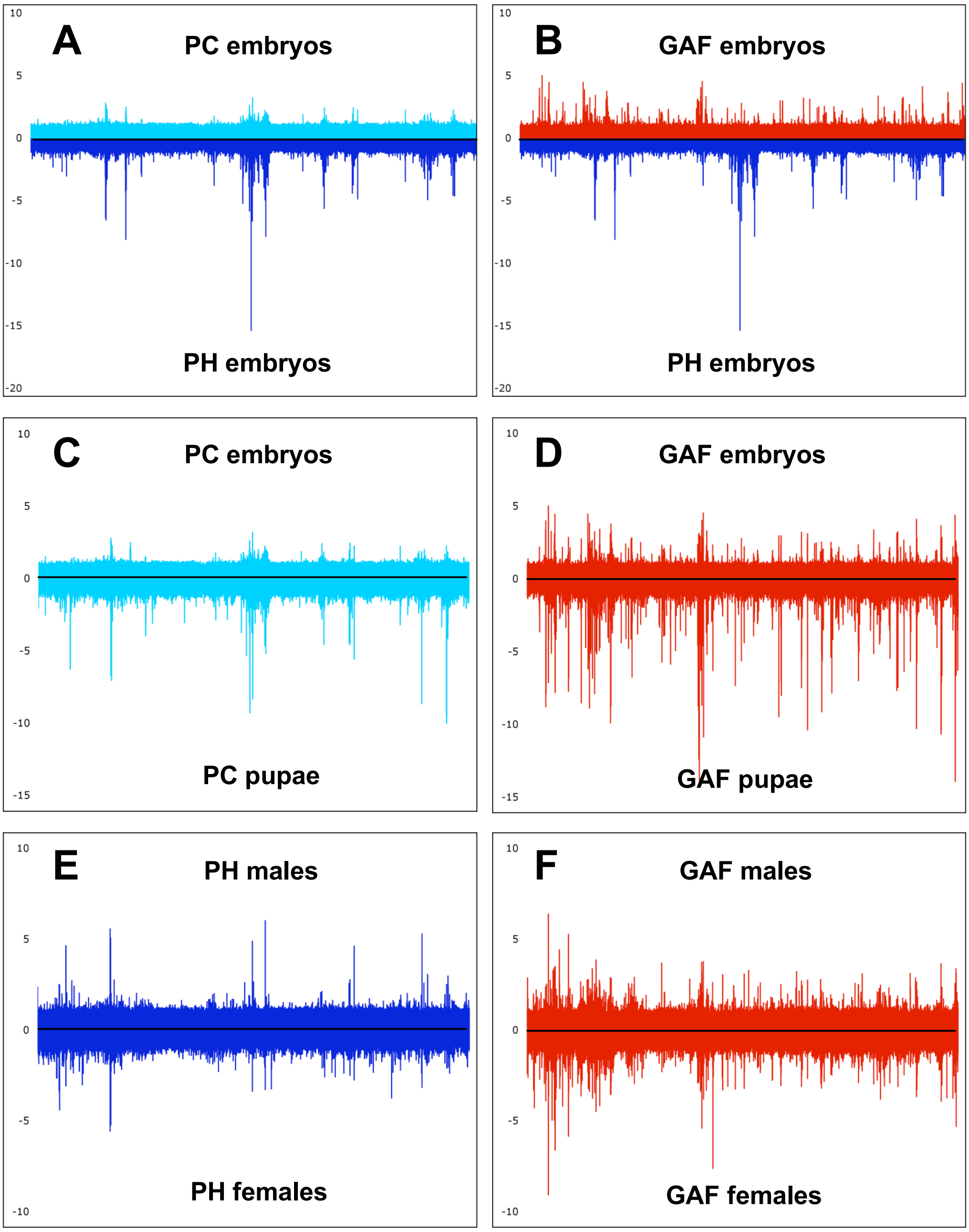

A
PC embryos
PH embryos
B
GAF embryos
PH embryos
C
PC embryos
PC pupae
D
GAF embryos
GAF pupae
E
PH males
PH females
F
GAF males
GAF females

Figure S5
