# Supplementary material for: Chromosomal Distribution of PcG Proteins during Drosophila Development
Source: PLoS Biol. 2006 Apr 20;4(6):e170. doi: 10.1371/journal.pbio.0040170 (PMC1440717; doi:10.1371/journal.pbio.0040170)
Supplement: Supplementary file 4 [file pbio.0040170.sg006.pdf]

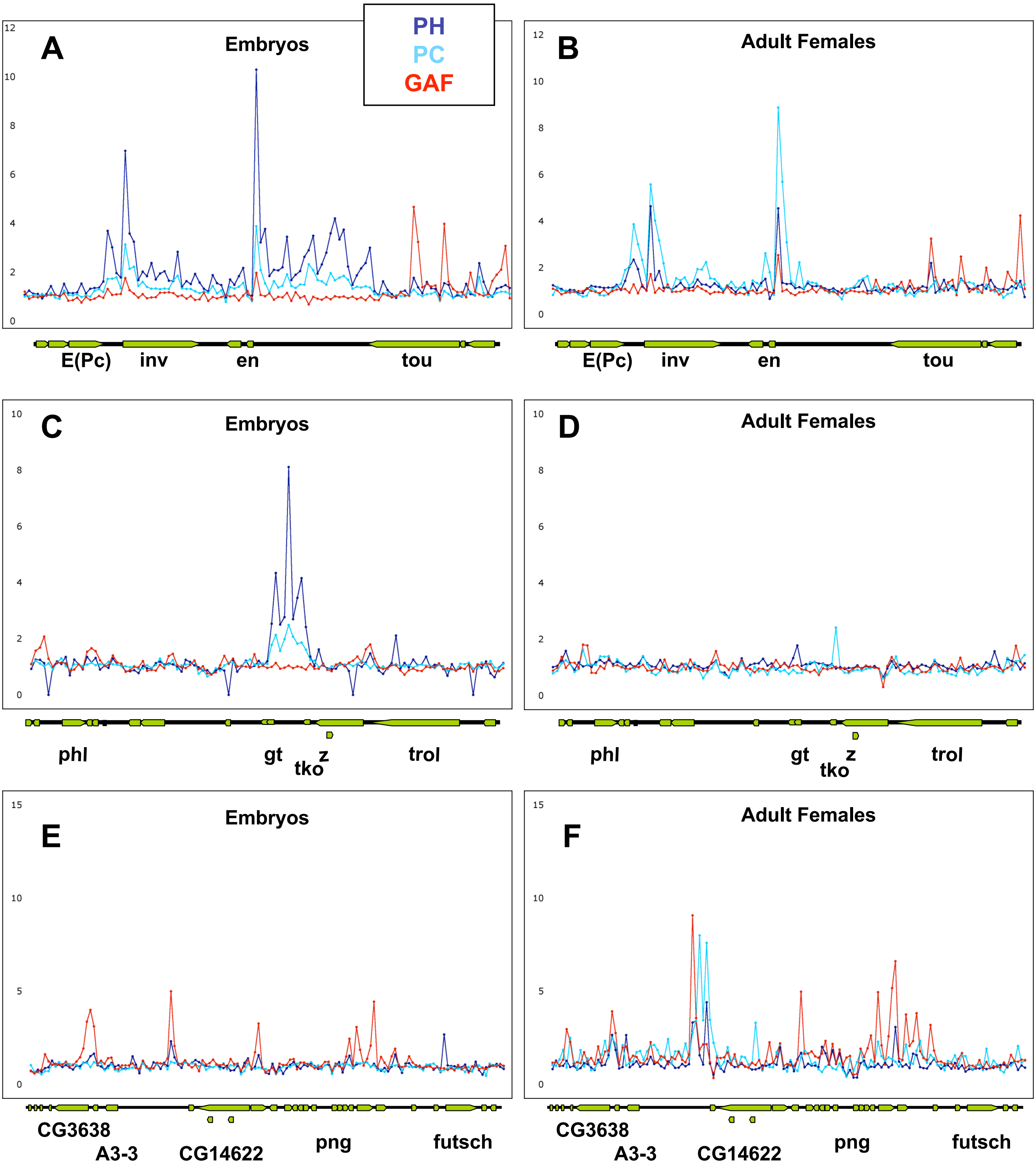

PH
PC
GAF
A
Embryos
12
10
8
6
4
2
0
E(Pc)
inv
en
tou
B
Adult Females
C
Embryos
phl
gt
tko
z
trol
D
Adult Females
E
Embryos
15
5
CG3638
A3-3
CG14622
png
futsch
F
Adult Females

Figure S6
