# Supplementary material for: Chromosomal Distribution of PcG Proteins during Drosophila Development
Source: PLoS Biol. 2006 Apr 20;4(6):e170. doi: 10.1371/journal.pbio.0040170 (PMC1440717; doi:10.1371/journal.pbio.0040170)
Supplement: Supplementary file 5 [file pbio.0040170.sd001.doc]

#### ***Protocol S1. Supporting Information on the Assembly of Montpellier-Yale Microarrays***

The genomic *Drosophila* DNA microarray used in this study combine a tiling path containing 2.9 Mb of the *Adh* region on the left arm of Chromosome 2 (2L) [40] with a tiling path covering 7 Mb of the tip of the X Chromosome. Several smaller genomic regions of interest and control sequences such as known PREs of the BX-C, the *ph* and *en* genes were also included in the array. The tiling paths were built by producing overlapping PCR fragments. In the Montpellier assembly, oligo pairs were designed for PCR amplification of DNA fragments of an average size of 1.9 kb using genomic DNA as a template. The neighboring PCR fragments have an overlap window of 0-100 bp. Repeated sequence elements were not masked. 4,153 high quality PCR fragments were obtained, covering the distal part of the X Chromosome (from coordinate 778,383 to 7,828,607 of the whole genome sequence release 4; this region includes 695 genes), the *scalloped* (*sd*) locus (X Chromosome, 137 kb), the *engrailed*/*invected* locus (*en*/*inv*; Chromosome 2R, 197 kb), the *hedgehog* (*hh*) locus (Chromosome 3R, 83 kb), the *cubitus interruptus* (*ci*) region (Chromosome 4, 71 kb) and control sequences in the BX-C (Chromosome 3R, 45 kb) including known PREs as *Fab-7*, *Mcp* and *bxd* as well as the first and last exons of the *Abd-B*, *abdominal-A* (*abd-A*) and *Ultrabithorax* (*Ubx*) genes. To this assembly, we added the Yale set described in Sun et al. [40]. The Yale set consists of a tiling path made of 3,648 PCR products, ranging in size from 850 to 920 bp and covering 2.9 Mb of the *Adh* region on Chromosome 2L (from coordinate 13,506,251 to 16,457,628 of the whole genome sequence release 4, including 255 genes), and of 192 fragments ranging from 430 to 500 bp and spanning 85 kb of a region located in 82F on Chromosome 3R.
